# Restricting child-directed ads is effective, but adding a time-based ban is better: evaluating a multi-phase regulation to protect children from unhealthy food marketing on television

**DOI:** 10.1186/s12966-023-01454-w

**Published:** 2023-05-26

**Authors:** Francesca R. Dillman Carpentier, Fernanda Mediano Stoltze, Marcela Reyes, Lindsey Smith Taillie, Camila Corvalán, Teresa Correa

**Affiliations:** 1 Hussman School of Journalism and Media, University of North Carolina, Chapel Hill, NC 27599 USA; 2 Carolina Population Center, University of North Carolina, Chapel Hill, NC 27516 USA; 3 Institute of Nutrition and Food Technology, University of Chile, Santiago, 7830490 Chile; 4 Department of Nutrition, Gilling’s School of Global Public Health, University of North Carolina, Chapel Hill, NC 27599-7400 USA; 5 School of Communication, Diego Portales University, Santiago, 8370067 Chile

**Keywords:** Childhood obesity, Children, Food advertising, Food marketing, Health policy, Television

## Abstract

**Background:**

As childhood obesity rates continue to rise, health organizations have called for regulations that protect children from exposure to unhealthy food marketing. In this study, we evaluate the impact of child-based versus time-based restrictions of “high-in” food and beverage advertising in Chile, which first restricted the placement of “high-in” advertisements (ads) in television attracting children and the use of child-directed content in high-in ads and, second, banned high-in ads from 6am-10pm. “High-in” refers to products above regulation-defined thresholds in energy, saturated fats, sugars, and/or sodium. High-in advertising prevalence and children’s exposure to high-in advertising are assessed.

**Methods:**

We analyzed a random stratified sample of advertising from two constructed weeks of television at pre-regulation (2016), after Phase 1 child-based advertising restrictions (2017, 2018), and after the Phase 2 addition of a 6am-10pm high-in advertising ban (2019). High-in ad prevalence in post-regulation years were compared to prior years to assess changes in prevalence. We also analyzed television ratings data for the 4–12 year-old child audience to estimate children’s ad exposure.

**Results:**

Compared to pre-regulation, high-in ads decreased after Phase 1 (2017) by 42% across television (41% between 6am-10pm, 44% from 10pm-12am) and 29% in programs attracting children (*P* < 0.01). High-in ads further decreased after Phase 2, reaching a 64% drop from pre-regulation across television (66% between 6am-10pm, 56% from 10pm-12am) and a 77% drop in programs attracting children (*P* < 0.01). High-in ads with child-directed ad content also dropped across television in Phase 1 (by 41%) and Phase 2 (by 67%), compared to pre-regulation (*P* < 0.01). Except for high-in ads from 10pm-12am, decreases in high-in ads between Phase 1 (2018) and Phase 2 were significant (*P* < 0.01). Children’s high-in ad exposure decreased by 57% after Phase 1 and by 73% after Phase 2 (*P* < 0.001), compared to pre-regulation.

**Conclusions:**

Chile’s regulation most effectively reduced children’s exposure to unhealthy food marketing with combined child-based and time-based restrictions. Challenges remain with compliance and limits in the regulation, as high-in ads were not eliminated from television. Yet, having a 6am-10pm ban is clearly critical for maximizing the design and implementation of policies that protect children from unhealthy food marketing.

## Background

Childhood obesity and obesity-related disease are increasing worldwide, with outcomes such as diabetes, cardiovascular disease, cancers, and psychological comorbidities creating a global burden with social and economic implications beyond rising health care costs [1, 2]. Childhood obesity has overtaken undernourishment for school-aged youth in some parts of the world [1, 3]. Countries in Latin America are among the nations with the highest rates of childhood obesity [4–6].

Unhealthy food marketing, of which television advertising is an important component, contributes to this growing burden [7–9]. Children’s exposure to unhealthy food and beverage advertisements (food ads henceforth) is associated with higher consumption of the advertised foods and general increased caloric intake [10–14]. Global health organizations have thus called for statutory regulations to reduce unhealthy food marketing to children [15–18]. Countries seeking to develop such regulations [6, 19, 20] are looking to Chile’s comprehensive Food Labeling and Advertising Law [21], which attempts to curb childhood obesity via school food sales, front-of-package warning labels, and marketing restrictions for qualifying foods and beverages. Described in detail elsewhere [22, 23], products in Chile are subject to regulation if they exceed defined nutrient thresholds in (henceforth are “high in”) energy, saturated fats, sugars, and sodium. Nutrient thresholds were gradually raised across three phases beginning June 2016 and arriving June 2019 at the full qualifications for identifying “high-in” products warranting restriction.

**Fig. 1:**
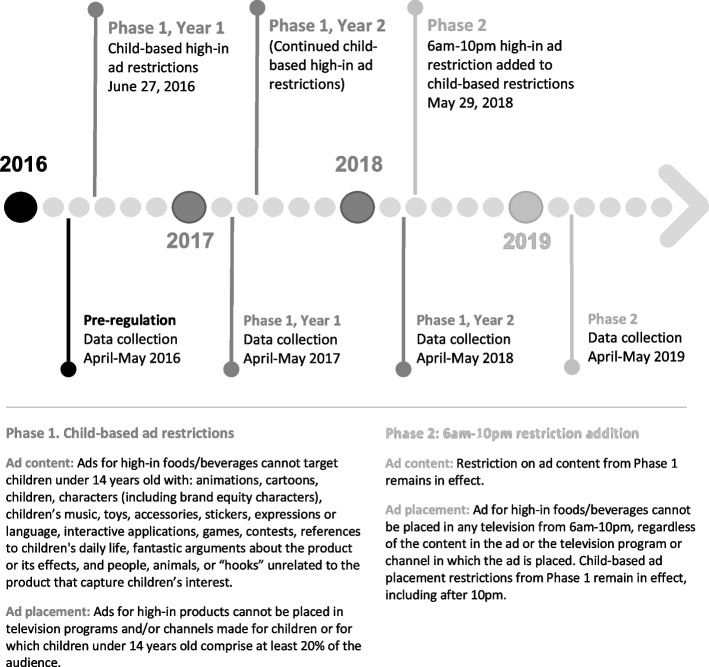
Timeline of regulatory phases and months of television sampling to examine pre-regulation (2016), two years of post-regulation during Phase 1 (2017, 2018), and one year after Phase 2 (2019). “High-in” refers to products above regulation-defined thresholds in energy, saturated fats, sugars, and/or sodium according to Chile’s Food Labeling and Advertising Law [21]

Marketing restrictions were implemented in two phases (See Fig. 1). Beginning in June 2016, Chile banned advertising high-in products in media made for children under 14 years old or in programs for which children comprised at least 20% of the audience (programs attracting children henceforth), a measure similar to children’s television advertising restrictions found in other countries [24]. The use of child-directed appeals in the advertising content, including child actors, cartoon characters, toys, games, or play (child-directed ad content henceforth) [25] was also banned from high-in product marketing. An early study of this first phase on television advertising [25, 26] found that high-in ads in total, in programs attracting children, and with child-directed ad content decreased significantly from the months before implementation to the year following implementation. This early study qualified high-in foods based on the introductory nutrient thresholds from the first phase, which were designed to ease industry into the intended nutritional standards [25]. The second phase further banned any advertising of high-in products across all television from 6am-10pm starting June 2018. The progression from child-based restrictions in Phase 1 to both child-based and time-based television restrictions in Phase 2 positions Chile’s law as the most robust regulation of food advertising to date [27] and potentially the most promising statutory measure for reducing children’s exposure to unhealthy food advertising.

In this study, we assess the progressive impact of Chile’s multi-phased regulation on both the prevalence of high-in ads on television and children’s exposure to these ads from 2016 to 2019, with “high-in” denoting products excessive in energy, sodium, sugars, and/or saturated fats based on final 2019 thresholds. By assessing the different phases of Chile’s law, we are able to evaluate the effectiveness of a stepwise statutory policy implementation that begins with child-based advertising restrictions, such as those adopted in other countries [24], and adds a broader 6am-10pm ban on high-in food and beverage television advertising. This study is therefore critical to informing policy development by disentangling effects of different policy actions [24, 28, 29] and adding to the scant body of research on governmental efforts to reduce food marketing to children [27, 30–33].

## Method

### Sample

Chile’s National Television Council provided recordings of all television programming shown from 6am-12am in April–May 2016 through 2019 on Chile’s four main broadcast channels (TVN, C13, CHV, Mega) and four cable channels (Disney Channel, Discovery Kids, Cartoon Network, Fox) with the highest audience ratings among children 4–12 years old and teens 13–17 years old according to Kantar Ibope Media ratings provided by the Television Council [34]. A stratified random sample of two constructed weeks of programming was drawn for each year to account for content variation across different days of the week [35]. That is, for each year sampled, one Sunday was randomly selected from the weeks comprising April, one Monday was randomly selected from the same weeks, one Tuesday, and so on, until all 7 days were represented for the month of April. The same procedure was done to select a random Sunday, Monday, Tuesday, etc., in May for the given year. As these months in Chile include Easter observances, samples in each year were checked for the presence of ads relating to festivals or holidays for potential impact on advertising and were found to be negligible (1% or fewer food ads in first three years and 7% of food ads in the last year consisting primarily of non-regulated grocery ads).

All ads with an easily seen and recognizable food or beverage (excluding alcohol and nutritional supplements) in each of the 8 channels between 6am-12am on selected days were analyzed. This includes ads for non-food companies that featured a product in the ad and excludes ads, including brand-only ads, that had no products shown or easily visible. No Institutional Review Board approval was needed, as no human subjects were involved.

### Procedure

Each year from July–September, between 7 and 8 trained coders in Chile (6 remained for all 4 years) documented food ads and analyzed their content against a codebook, presented elsewhere [25], that identified child-directed ad content defined in the regulation. The codebook was pretested each year with a subsample of ads by two trained coders recruited from the communication school at Diego Portales University. All codes reached acceptable levels of intercoder reliability (Cohen’s Kappa > 0.70).

Trained nutritionists at the University of North Carolina at Chapel Hill were given a list of products appearing in each sampled ad (up to 4 per ad in 2016, up to 7 in 2017–2019) and linked these products to Nutrition Facts Panel data collected by the University of Chile’s Institute of Nutrition and Food Technology according to guidelines by INFORMAS (International Network for Food and Obesity/Non-communicable Diseases Research, Monitoring and Action Support) [36]. Products were classified as under or over thresholds in energy, saturated fats, sugars, and sodium defined in the final phase implemented in June 2019. Solid food thresholds were 275 kilocalories, 400 mg sodium, 10 g sugars, and/or 4 g saturated fats per 100 g. Liquid thresholds were 70 kilocalories, 100 mg sodium, 5 g sugars, and 3 g saturated fats per 100 ml. Previously used data from 2016 and 2017 [25, 37] were refined to improve product identification and nutritional data matching.

Product classifications were linked to each sampled ad and matched with its content analysis, program and time in which the ad was shown, and the program’s television rating for the 4–12 year-old child audience segment. Despite the focus of the regulation on children under age 14, television ratings were only available in the aggregate for audience segments covering ages 4 to 12 years and ages 13 to 17 years. To calculate 4–12 year-old children’s exposure to these ads, we used daily gross rating points (GRP), derived as the sum of television audience ratings across ads within a given product category (e.g., high-in beverage, non-high-in beverage) for one day. For broadcast television, the ad audience rating was based on the rating of each ad’s host program. For cable television, the ad audience rating was based on the daily rating of each ad’s cable channel, as we did not obtain audience ratings disaggregated by program. GRP is a standard metric of advertising exposure. One GRP indicates the given type of ad reached 1% of 4–12 year-old children. Two GRP may indicate reaching 1% of the child audience twice or 2% of the child audience once. About 3.6 million 0–14 year-old children live in Chile [38].

### Codes

#### High-in advertising

We used the final and most stringent nutrient thresholds implemented in the final phase (June 2019) to categorize products. In line with how Chile’s regulation would identify an ad for restriction, any food ad with at least one product above any threshold was coded as “1” for high-in ad. Ads without any products above a threshold were coded as “0.” Ads were not given a high-in code if none of the products within the ad could be identified, as these products could not be matched with nutritional data.

#### Programs attracting child audiences

Given that Chile’s first regulation was in effect for television sampled in 2017, 2018, and 2019, ads in all years of analysis were coded as “1” if they were placed in television programs that were listed by program producers as being made for children and/or had a minimum 20% child audience composition based on television ratings of total and 4–12 year-old child audiences. Ads in programs not meeting either criterion were coded as “0.”

#### Child-directed ad content

Aligning with Chile’s regulation, ads were coded for the presence of child figures or voices; licensed and brand equity characters; animations or cartoons; animals, anthropomorphized objects, and other characters interesting to children; celebrities, athletes, people, promotional gifts, prizes, contests, or interactive games of interest to children; and references to fantasy, magic, school, play, popular child phrases, and child life. Ads featuring any one of these strategies were coded as “1” for being child-directed, “0” for absence of these strategies.

#### Time-based advertising

Chile´s second regulation phase, implemented June 2018, banned all high-in food ads from 6am to 10pm, regardless of their program placement or content. This phase was in effect for the 2019 TV sample. Any food ad placed in television programs aired from 6am to 10pm was coded as “1.” Ads aired from 10 pm to 6 am were coded as “0.”

### Analysis

Frequencies and percentages were used to describe weekly prevalence of high-in ads in total, high-in ads featuring child-directed ad content, high-in ads in programs attracting child audiences, high-in ads aired between 6am-10pm, and high-in ads aired between 10pm-12am. Percentages were based on all food ads matched with nutritional data. Two types of comparisons were made. First, 2016 pre-regulation was treated as a baseline measure compared against each subsequent year. Percentage change was calculated using the formula (YearX—Year1)/Year1, with Year1 being the 2016 pre-regulation data and YearX being a subsequent year, e.g., 2019. Second, adjacent years were compared to examine change from one year to the next. Percentage change was similarly calculated with formula [Year(X + 1) – YearX]/YearX to examine how one year, e.g., 2018 as YearX, changed in relation to the next year, e.g., 2019 as Year(X + 1). Changes between years were tested for statistical significance using Pearson chi-square tests of independence evaluated at *P* < 0.01 to control for error inflation.

To assess change in children’s overall exposure to high-in food advertising, 4–12 year-old children’s average daily gross rating points (GRP) for high-in ads in total were described for each year analyzed. Differences between years were evaluated using percentage change and chi-square tests described above. Also evaluated were changes in children’s exposure to high-in ads placed in television attracting child audiences, given the aforementioned focus of policies across the globe on restricting food advertising in children’s television [24]. Finally, descriptives were used to explore the food categories comprising the largest numbers of high-in ads per year to identify shifts in the types of high-in products promoted. Percentages of high-in ads per product category were out of the total high-in ads for the given year. Analyses were conducted in Microsoft Excel version 16.67 and SPSS version 27.

## Results

### Changes in high-in ad prevalence from pre-regulation

As Table 1 shows, the most dramatic changes in ad prevalence were seen between pre-regulation and Phase 2. The total number of weekly food ads decreased by 14% from 2016 pre-regulation to 2019 after the Phase 2 6am-10pm ban. More important, the weekly number of high-in ads in total dropped by 64% from 2016 levels to 2019. Whereas 70% of the food ads in 2016 were for high-in products, only 29% of the food ads in 2019 were for high-in products. Also, the number of high-in ads shown between 6am-10pm and between 10pm-12am dropped from 2016 pre-regulation to 2019 post-Phase 2 by 66% and 56%, respectively. This 10pm-12am drop is particularly notable because high-in ads were allowed after 10pm as long as the ads themselves did not contain child-directed content. The table also shows similarly large decreases in the number of high-in ads with child-directed ad content (by 67%) and high-in ads placed in programs attracting child audiences (by 77%) from 2016 to 2019.

**Table 1:** Frequencies and percentages of food and beverage advertisements found across one week of Chilean television

**Type of Food/Beverage Advertisement**	**2016** **(pre-regulation)** **N**	**2017** **(Phase 1 Year 1)** **N**	**2017 vs. 2016** **percentage** **change**	**2018** **(Phase 1 Year 2)** **N**	**2018 vs. 2016** **percentage** **change**	**2018 vs. 2017** **percentage** **change**	**2019** **(Phase 2 Day Ban)** **N**	**2019 vs. 2016** **percentage** **change**	**2019 vs. 2018** **percentage** **change**
All ads	2845	2805	-1.41 *P* = 0.595	3145	**10.54** *P* < 0.001	**12.12** *P* < 0.001	2423	**-14.83** *P* < 0.001	**-22.96** *P* < 0.001
Ads matched with nutritional data	2779	2545	**-8.42** *P* < 0.01	2814	1.26 *P* = 0.640	**10.57** *P* < 0.001	2391	**-13.96** *P* < 0.001	**-15.03** *P* < 0.001
(% of all ads)	(97.68%)	(90.75%)	**-7.09** *P* < 0.001	(89.49%)	**-8.38** *P* < 0.001	-1.39 *P* = 0.106	(98.66%)	1.00 *P* = 0.011	**10.25** *P* < 0.001
High-in ads in total	1948	1136	**-41.68** *P* < 0.001	1237	**-36.50** *P* < 0.001	8.89 *P* = 0.038	697	**-64.22** *P* < 0.001	**-43.65** *P* < 0.001
(% of ads matched)	(70.11%)	(44.62%)	**-36.36** *P* < 0.001	(43.96%)	**-37.30** *P* < 0.001	-1.48 *P* = 0.627	(29.14%)	**-58.44** *P* < 0.001	**-33.71** *P* < 0.001
High-in in programs attracting child audiences	535	382	**-28.60** *P* < 0.001	291	**-45.61** *P* < 0.001	**-23.82** *P* < 0.001	123	**-77.01** *P* < 0.001	**-57.73** *P* < 0.001
(% of ads matched)	(19.25%)	(15.01%)	**-22.03** *P* < 0.001	(10.34%)	**-46.29** *P* < 0.001	**-31.11** *P* < 0.001	(5.15%)	**-73.25** *P* < 0.001	**-50.19** *P* < 0.001
High-in with child-directed ad content	1493	879	**-41.13** *P* < 0.001	1015	**-32.02** *P* < 0.001	**15.47** *P* < 0.01	498	**-66.64** *P* < 0.001	**-50.94** *P* < 0.001
(% of ads matched)	(53.72%)	(34.52%)	**-35.74** *P* < 0.001	(36.07%)	**-32.86** *P* < 0.001	4.49 *P* = 0.237	(20.83%)	**-61.22** *P* < 0.001	**-42.25** *P* < 0.001
High-in between 6am-10pm (banned June 2018)	1593	935	**-41.31** *P* < 0.001	1019	**-36.03** *P* < 0.001	8.98 *P* = 0.057	540	**-66.10** *P* < 0.001	**-47.01** *P* < 0.001
(% of ads matched)	(57.32%)	(36.74%)	**-35.90** *P* < 0.001	(36.21%)	**-36.83** *P* < 0.001	-1.44 *P* = 0.689	(22.57%)	**-60.62** *P* < 0.001	**-37.67** *P* < 0.001
High-in between 10pm-12am	356	201	**-43.54** *P* < 0.001	218	**-38.76** *P* < 0.001	8.46 *P* = 0.406	157	**-55.90** *P* < 0.001	**-27.98** *P* = 0.020
(% of ads matched)	(12.79%)	(7.88%)	**-38.39** *P* < 0.001	(7.75%)	**-39.41** *P* < 0.001	-1.65 *P* = 0.841	(6.57%)	**-48.63** *P* < 0.001	**-15.23** *P* = 0.101

High-in refers to ads with at least one product exceeding one or more of the following thresholds: 275 kilocalories energy, 400 mg sodium, 10 g sugars, 4 g saturated fats per 100 g solid foods; 70 kilocalories energy, 100 mg sodium, 5 g sugars, 3 g saturated fats per 100 ml liquids. Comparisons made within row only, using Pearson chi-square tests. Percentage change is based on change from preceeding year calculated as (Year 2 – Year 1)/Year1. Bold comparisons are significant at *P* < 0.01

To a lesser extent, high-in ads across all categories analyzed dropped significantly (*P* < 0.001) when comparing 2016 pre-regulation with 2017 Phase 1, Year 1 and when comparing 2016 with 2018 Phase 1, Year 2, when child-based restrictions were in place. To highlight some figures from Table 1, the number of high-in ads placed in programs attracting child audiences dropped from 2016 pre-regulation levels by 29% in 2017 and by 46% in 2018. The number of high-in ads across programs that used child-directed ad content dropped from 2016 levels by 41% in 2017 and by 32% in 2018.

### Changes in high-in ad prevalence between adjacent years

When comparing the first and second years of Phase 1 (2017 and 2018), which had the same restrictions in place, the total number of food ads increased (*P* < 0.001) but the total number of high-in ads did not (*P* = 0.038). However, within high-in ads, the number of high-in ads using child-directed ad content increased (*P* < 0.001) from 2017 to 2018, whereas the number of high-in ads placed in programs attracting child audiences decreased (*P* > 0.001). That is, within Phase 1 years, high-in ads with child-directed content increased primarily in programs that were not considered to attract children. Regardless of this increase within Phase 1, all high-in ad categories were reduced in 2017 and in 2018 when compared to 2016 pre-regulation.

An examination of differences between 2018 Phase 1 and 2019 Phase 2 indicates a significant decrease in high-in advertising in total, between 6am-10pm, in programs attracting children, and with child-directed ad content, as well as a significant decrease in food ads overall (*P* < 0.001). The number of high-in ads shown between 10pm-12am was statistically equivalent between 2018 and 2019, although the difference between these two years—a 28% decrease—approached statistical significance (*P* = 0.020). See Table 1 for a full report, including the final number of ads with products matched with nutritional data between 2016 and 2019.

### Changes in children’s exposure to high-in ads

**Fig. 2:**
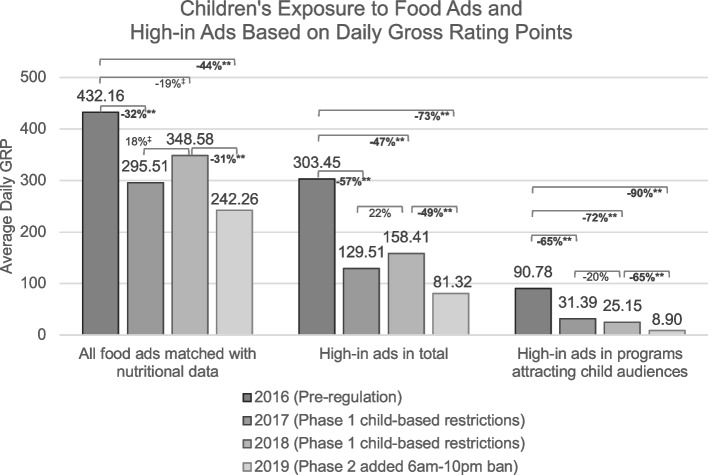
Bars represent average daily gross rating points (GRP) for 4–12 year-old child audiences for food/beverage ads (matched with nutritional data) in total, high-in ads in total, and high-in ads placed in TV programs attracting child audiences for samples of television from April–May 2016 pre-regulation and April–May 2017, 2018, and 2019 post-regulation. GRP is a standard industry metric, in which 1 GRP indicates the given type of ad reached 1% of the target audience and 2 GRP may indicate reaching 1% of the target audience twice or 2% of the target audience once. Final (June 2019) regulation thresholds in energy, saturated fats, sugars, and sodium are used to define “high-in” products across all years. Differences in amounts of ads within each category are tested with Pearson chi-square tests evaluated at *P* < 0.01. Percentage change reflects change relative to either pre-regulation or to prior year and calculated as (Year 2 – Year 1)/Year 1. **P < 0.001; *P < 0.01; ^‡^ = *P* < 0.05, approaches significance

Changes in children’s exposure to high-in ads are illustrated in Fig. 2. Average daily GRP of 4–12 year-old child audience for food ads in general, for high-in ads in total, and for high-in ads placed in programs attracting children are shown for each year, in addition to the percentage changes from 2016 pre-regulation to subsequent years and between subsequent years. GRP for high-in ads in total and in programs attracting children were significantly lower than 2016 pre-regulation levels for all post-regulation years (*P* < 0.001). From pre-regulation levels, GRP for high-in ads in total fell by 73% to reach a level below 100 by 2019 post-Phase 2 (81 GRP) for high-in ads in total. A GRP of 100 can be interpreted as either of the following: 100% of children in the television audience were exposed to 1 high-in ad per day or fewer than 100% of children received multiple exposures to equal the equivalent number of impressions as 1 exposure per child. At pre-regulation, daily GRP was 303 for high-in ads in total, which constituted about 70% of the exposure to food ads generally (432 GRP in 2016).

At pre-regulation, 21% of food ad exposure, and 30% of high-in ad exposure, was for high-in ads placed inside programs attracting children (91 GRP). This level decreased by 65% beginning with Phase 1 and then decreased by another 65% between the second year of Phase 1 (2018) to 2019 post-Phase 2 to reach a low of 9 GRP. This near-zero daily GRP in 2019 for high-in ads within programs attracting children represents a drop of 90% from pre-regulation levels. Findings also show continued children’s exposure to the remaining high-in ads on television. As seen in Fig. 2, the average daily GRP for food ads in 2019 post-Phase 2 was 242, and 34% of that exposure (81 GRP) was for high-in ads.

### Changes in the types of high-in products advertised

To explore what types of products were being represented in high-in advertising at each phase, the most prevalent food categories within high-in ads were identified for each sampled year. Findings are shown in Table 2. At pre-regulation (2016), high-in products from sodas (16% of high-in ads), sweets and non-grain-based desserts (16% of high-in ads), and meat, poultry and meat substitutes (11% of high-in ads) were the most prevalent food categories. In Phase 1 years (2017, 2018), a shift occurred such that the prevalence of sodas (2% and 5% of high-in ads in 2017 and 2018, respectively) and sweets (3% and 10%, respectively) was overtaken by the prevalence of fast foods (21% and 27%) and dairy products and dairy substitutes (23% and 12%). At Phase 2 (2019), the most frequently advertised food products remained fast foods (30%) and dairy products and substitutes (16%). A cursory qualitative examination of the fast-food category showed that many of these ads were for food delivery services, with foods from a variety of food establishments (e.g., restaurants, supermarkets) shown.

**Table 2:** Changes in weekly number of ads with at least one high-in product based on food category

Food category	2016	2017	2018	2019
	N (%)	N (%)	N (%)	N (%)
**Sodas**	**646 (16.04%)**	57 (2.54%)	154 (5.42%)	19 (1.4%)
**Sweets and Non–grain-based Desserts**	**631 (15.67%)**	67 (2.98%)	**291 (10.25%)**	117 (8.5%)
**Meat, Poultry and Meat Substitutes**	**448 (11.12%)**	**308 (13.71%)**	231 (8.14%)	**157 (11.3%)**
**Fast Foods**	429 (10.65%)	**467 (20.78%)**	**753 (26.52%)**	**413 (29.8%)**
Salty Snacks	386 (9.59%)	62 (2.76%)	75 (2.64%)	117 (8.5%)
**Dairy Products and Dairy Substitutes**	340 (8.44%)	**507 (22.56%)**	**327 (11.52%)**	**221 (16.0%)**

The three largest categories per year are in bold. Percentages are based on the number of weekly high-in ads in that year

## Discussion

This study is a direct evaluation of Chile’s stepwise phased approach to the implementation of a statutory policy aimed at restricting unhealthy food marketing to children. Assessments included the extent to which advertising of foods and beverages high in energy, sugars, saturated fats, and/or sodium were reduced across Chilean television programs and channels. This study also evaluated how this phased approach reduced children’s exposure to unhealthy food advertising. To review, the first phase introduced child-based restrictions on high-in ad content and placement and the second phase added a 6am-10pm ban on high-in ads across television. Nutrient thresholds defining “high-in” products began with introductory levels to gradually ease industry toward final thresholds implemented in a third phase. Although all thresholds were published by 2015 [23], allowing industry to adjust products and marketing early to comply with final restrictions, industry was only required to comply with the lower thresholds in place during prior phases. Given the final thresholds constitute the regulatory goal for qualifying products for restriction, this study evaluates unhealthy food advertising using the final thresholds.

We found gradual decreases in high-in food advertising across phases. Lowest levels of high-in advertising were reached after Phase 2 was implemented, with a 64% decrease in high-in ads from pre-regulation amounts. This decrease was seen between 6am-10pm and between 10pm-12am, with high-in advertising decreasing by 66% and 56% from pre-regulation, respectively, presumably due to the combination of Phase 2’s 6am-10pm ban and continued child-based restrictions after 10pm. High-in sodas and sweets, the two most prevalent product categories promoted in high-in ads at pre-regulation, were rarely promoted in post-regulation years.

Children’s exposure to high-in ads likewise decreased, according to television audience gross rating points (GRP) by 73% overall from pre-regulation levels. However, exposure was not eliminated.

Despite the added 6am-10pm restriction in Phase 2, 29% of food ads on television promoted a high-in product and 34% of children’s daily GRP to food ads consisted of exposure to those high-in ads. This is a smaller amount of exposure than pre-regulation study projections, which estimated children’s 4-12y GRP for high-in ads would be 229 per day by the last regulation phase, comprising 51% of total food ad GRP if advertising did not change (Phase 2 GRP was 81 points) [37]. These findings also show improvement from an earlier study of preschool and adolescent children in Chile, which found viewing minutes of high-in ads decreased by 44% and 58%, respectively, from 2016 pre-regulation to 2017 one year after Phase 1 [26]. Thus, it is clear the addition of the 6am-10pm ban significantly improved upon the effectiveness of the child-based restrictions, although some high-in ad exposure remained.

Findings suggest a child-based restriction is perhaps most effective in eliminating high-in ads placed in children’s programming, rather than high-in ads using child-directed ad content. That is, high-in ads placed in programs attracting children fell significantly in each year after pre-regulation to result in an overall 77% drop by Phase 2, compared to pre-regulation levels. Children’s exposure to high-in ads in these programs was near zero by Phase 2, although placement in these programs only constituted 30% of children’s exposure to high-in ads at pre-regulation. These findings are comparable to those in South Korea, which banned advertising of foods high in energy, saturated fats, sugars, or sodium from both children’s television and television aired between 5pm-7pm, decreasing children’s exposure to those ads by 82% in the restricted programs [33].

The continued presence of high-in advertising outside of children’s programming throughout the post-regulation period underscores the importance of restricting child appeals in the ad content, given children are drawn to ads with characters, toys, and the like [9]. We found that high-in ads using child-directed marketing appeals in the ad content decreased as intended after Phase 1 was implemented but increased in the second year of Phase 1, albeit this level was still significantly lower than pre-regulation levels. This increase within Phase 1 was unexpected and might reflect the rise we found in food delivery service ads, which promote a transportation service featuring foods from a variety of sources. Perhaps these delivery companies, new to the food sector, did not have the level of understanding of the regulation that food manufacturers, distributors, and vendors would have had by this point. It is also possible the food industry was testing limits of the definition of “child-directed” with the use of content or figures that might be considered of general appeal if the regulatory definition was not strictly applied. In any case, high-in ads with child-directed ad content then decreased in Phase 2 to a level that was 51% lower than pre-regulation levels, demonstrating the benefit of adding a 6am-10pm restriction on high-in advertising. Yet, high-in ads with child-directed ad content still comprised 21% of the smaller number of high-in ads remaining at Phase 2. Additional research is needed to understand why child-directed marketing appeals continued to be present through each phase.

Television has been the primary advertising medium in Chile [39], although we should note that food companies are increasingly using online marketing to promote their products [40]. Thus, it is important to understand how the food marketing environment on television might change in response to a gradual implementation of goals that include guidance around unhealthy product qualifications, ad content, and ad placement. For instance, one might anticipate a shift to promoting unhealthy foods at night in response to a 6am-10pm ban. We did not observe this type of shift, which is especially relevant given Chile’s National Television Council (2020) [34] reported that 4–12 year-old children did most of their television viewing between 8pm-11pm in 2019 when Phase 2 was implemented [34]. We did observe that companies took the opportunity to gradually reduce the advertising of high-in products in general audience programs, which was allowed during Phase 1 as long as the ads themselves did not feature appeals defined in the regulation as child-directed. We also observed the largest drops in high-in advertising frequency and exposure within television programs attracting child audiences, although both companies’ ad placement decisions and changes we observed in exposure are at least partly affected by an overall drop in audience for these programs across the sampled time period as documented by Chile’s National Television Council [34]. Finally, we observed a low but continued level of children’s exposure to the high-in ads that persisted in Phase 2 television.

These findings highlight the successes of Chile’s food marketing policies as a means of protecting children from unhealthy food marketing. These findings also support the evidence showing that statutory food policies can effectively protect children from food marketing exposure and power [29] and are more likely to be effective when comprehensive in restricting marketing content and placement *wherever* and *whenever* children might be exposed [29]. Mixed results have been found among the few studies evaluating the impact of other statutory food marketing regulations, which vary in the foods, places, and content restricted, in addition to the geographic scope of restriction [27–29]. Perhaps the most comparable with Chile’s regulation, food advertising regulations in the United Kingdom were implemented with a stepwise approach that showed partial effectiveness after partial implementation and greater success after full implementation. In 2007, a content restriction banned the use of content appeals attractive to children in television ads for foods high in saturated fats, sugars, and sodium on children´s airtime and a scheduling restriction banned those ads in or around programs with child audiences [41]. Six months after the scheduling restriction was implemented, children´s exposure to the banned food ads as a proportion of all food ads increased [30]. After two years of implementation, food ads with appeals banned by the regulation had decreased significantly during children’s airtime but increased substantially overall and during adult airtime compared to pre-regulation [41].

We therefore argue that banning child-directed appeals in all high-in ad content, rather than just in high-in ads aired during children´s airtime [42], is a strength of the Chilean regulation and an important strategy for addressing the full spectrum of television viewed by children. This strategy addresses the World Health Organization's call to countries to reduce children’s exposure to unhealthy food marketing and the persuasive strategies used to target them [16, 18] and also aligns with calls to reduce children's exposure to unhealthy food marketing regardless of the time, intended audience, or presence of adults in the audience [18, 43].

In this study, we used the final and most stringent nutrient thresholds not yet implemented at the time of data collection, given our goal was to assess the impact of adding a 6am-10pm ban to child-based regulations on reducing children’s exposure to unhealthy food advertising on television. Thus, the numbers of high-in ads reported here are higher than the actual prevalence of high-in ads each year, according to the corresponding nutrient threshold. The percentages of high-in ads reported here should therefore not be interpreted as percentages of non-compliance with the regulations as they would be overestimated. Additional research is required to examine to what extent companies, television channels, and related stakeholders comply with these regulations over time.

Our study is also limited to describing child-directed marketing appeals in ad content based on the definition provided by the Chilean food marketing regulation. Although this regulatory definition is comprehensive [27], this definition excludes persuasive strategies that might not be exclusively child-directed yet still appealing to children [44]. Additionally, our results are constrained by the challenges of identifying creative content strategies and food products on television advertisements with dense audiovisual messages. We implemented several strategies to overcome this challenge, such as yearly coders’ training and intercoder agreement assessments to ensure the consistency of coding practices and reliability of the protocol. We have also improved our data cleaning and food product matching strategies over these four years.

We must also note that we did not identify whether advertising changes reported were associated with modifications to the brands' portfolio or product nutrient reformulation. Future research is needed to examine the underlying motives of advertising shifts in this regulated context. Finally, it must be emphasized that the decreases in high-in food ads found in this study only account for changes within the television channels sampled. We cannot generalize to high-in advertising frequencies in other media such as the internet, nor in spaces outside of media, such as outdoor advertising or marketing in public venues. An examination of digital media is especially relevant, given the likely migration of high-in ads from television to digital platforms [45], where children and adolescents spend most of their screen time [46, 47]. Future studies on the nature and extent of digital and extramedia food marketing in the Chilean regulated context are therefore critical to understand how effective Chile’s food marketing policy is when applied beyond television.

## Conclusions

Chile’s multi-phase food marketing regulation has been effective in reducing children’s exposure to unhealthy food advertising, with a 6am-10pm restriction on food advertising yielding significantly greater reductions beyond the effectiveness of child-based ad content and placement restrictions. However, the continued exposure children have to television outside of programs intended for children and programs shown at night highlights the importance of including child-based regulations that apply to all programming in addition to a time-based ban. The reductions in prevalence and exposure found in this study have major implications for informing the design of stepwise policies aimed at reducing children’s preferences for and consumption of obesogenic foods and beverages through the reduction of unhealthy food promotion. That is, policies aimed at protecting children from unhealthy food marketing exposure and power must include broadly defined content-, placement-, and time-based restrictions if they are to be effective in mitigating the potential harm unhealthy food marketing has on children.

## Data Availability

Datasets used for analysis are available upon reasonable request from the corresponding author.

## Abbreviation

GRP: Gross Rating Points
